# An inducible expression system for the manipulation of autophagic flux *in vivo*

**DOI:** 10.1080/15548627.2022.2135824

**Published:** 2022-10-30

**Authors:** Lars Schlotawa, Ana Lopez, Gentzane Sanchez-Elexpuru, Sylwia D. Tyrkalska, David C. Rubinsztein, Angeleen Fleming

**Affiliations:** a Department of Medical Genetics, University of Cambridge, Cambridge Institute for Medical, Research, Cambridge, UK; b Department of Physiology, Development and Neuroscience, University of Cambridge, Cambridge, UK; c UK Dementia Research Institute, University of Cambridge, Cambridge Institute for Medical Research, the Keith Peters Building, Cambridge, UK

**Keywords:** ATG4B, ATG5, autophagy, LC3-II, neurodegeneration, tamoxifen, zebrafish

## Abstract

Much of our understanding of the intracellular regulation of macroautophagy/autophagy comes from *in vitro* studies. However, there remains a paucity of knowledge about how this process is regulated within different tissues during development, aging and disease *in vivo*. Because upregulation of autophagy is considered a promising therapeutic strategy for the treatment of diverse disorders, it is vital that we understand how this pathway functions in different tissues and this is best done by *in vivo* analysis. Similarly, to understand the role of autophagy in the pathogenesis of disease, it is important to study this process in the whole animal to investigate how tissue-specific changes in flux and cell-autonomous *versus* non-cell-autonomous effects alter disease progression. To this end, we have developed an inducible expression system to up- or downregulate autophagy *in vivo*, in zebrafish. We have used a modified version of the Gal4-UAS expression system to allow inducible expression of autophagy up- or downregulating transgenes by addition of tamoxifen. Using this inducible expression system, we have tested which transgenes robustly up- or downregulate autophagy and have validated these tools using Lc3-II blots and puncta analysis and disease rescue in a zebrafish model of neurodegeneration. These tools allow the temporal control of autophagy via the administration of tamoxifen and spatial control via tissue or cell-specific ERT2-Gal4 driver lines and will enable the investigation of how cell- or tissue-specific changes in autophagic flux affect processes such as aging, inflammation and neurodegeneration *in vivo*.

**Abbreviations**: ANOVA: analysis of variance; Atg: autophagy related; Bcl2l11/Bim: BCL2 like 11; d.p.f.: days post-fertilization; Cryaa: crystallin, alpha a: DMSO: dimethyl sulfoxide; Elavl3: ELAV like neuron-specific RNA binding protein 3; ER: estrogen receptor; ERT2: modified ligand-binding domain of human ESR1/estrogen receptor α; Gal4: galactose-responsive transcription factor 4; GFP: green fluorescent protein; h.p.f.: hours post-fertilization; HSP: heat-shock protein; Map1lc3/Lc3: microtubule-associated protein 1 light chain 3; RFP: red fluorescent protein; SD: standard deviation; SEM: standard error of the mean; UAS: upstream activating sequence; Ubb: ubiquitin b

## Introduction

From *in vitro* studies, we have a detailed understanding of the intracellular regulation of autophagy. However, there remains a paucity of knowledge about how this process is regulated *in vivo*, particularly how changes in autophagic flux within different tissues affect the processes of development, aging and disease and whether non-cell autonomous effects play a role in these processes. Because upregulation of autophagy is a promising therapeutic strategy for the treatment of diverse disorders, including certain neurodegenerative and infectious diseases [1,2], it is vital that we understand how this pathway functions in different tissues in the context of the whole organism. Similarly, to understand the role of autophagy in the pathogenesis of disease, it is important to study this process in the whole animal to investigate how tissue-specific changes in flux and cell-autonomous *versus* non-cell-autonomous effects alter disease progression.

Typically, studies have used pharmacological upregulators or blocking agents to investigate how autophagy affects disease states *in vivo*. However, one limitation of this approach is the inability to manipulate autophagic flux with tissue- or cell-type specificity. Although up- or downregulation could be achieved by genetic means with the use of promoters to confer tissue specificity, these approaches typically lack the temporal control that is afforded by pharmacological agents. To overcome this, we have developed an inducible expression system to up- or downregulate autophagy *in vivo*, in zebrafish. We have used a modified version of the Gal4-UAS expression system to allow inducible expression of autophagy up- or downregulating transgenes. Gal4 is a transcriptional activator protein found in yeast which binds to its specific DNA recognition sequence upstream of the gene of interest, the upstream activation system (UAS). This two-component expression system was first established and is widely used in *Drosophila* [3] and has become a well-established technology in zebrafish [4]. We have developed our inducible system using a modified form of Gal4, namely ERT2-Gal4. ERT2-Gal4 comprises a modified ligand-binding domain (LBD) of human ESR1/estrogen receptor α fused to the Gal4 transcriptional activator. When no ligand is present, ESR (estrogen receptor) is bound and held in the cytoplasm by heat-shock proteins (HSPs), hence the Gal4 is also retained in the cytoplasm and cannot activate transcription. Ligand binding causes HSP-ER dissociation, thereby permitting nuclear translocation and transcriptional activity of the ER and with it, translocation of Gal4. This approach has previously been used for the temporal control of Cre recombinase (reviewed in ref [5].) and for temporal control of transcription factors [6]. Importantly, the synthetic ligand 4-hydroxy-tamoxifen (4-OHT, hereafter called tamoxifen) but not endogenous estrogens bind to the modified ERT2 [7]. Furthermore, tamoxifen does not cause toxicity in zebrafish embryos or larvae at the concentrations required to dissociate HSP/ER binding.

Using this inducible expression system, we have tested a panel of transgenes to find those that robustly up- or downregulate autophagy. Atg5 was considered as a candidate to upregulate autophagy. In mammalian cell cells, ATG5 conjugates with ATG12 and forms a complex with ATG16L1 that promotes the lipid conjugation of MAP1LC3/LC3 to form LC3-II, which is important for autophagic flux [8]. Overexpression of *atg5* in larvae of a zebrafish Parkinson disease model leads to upregulation of autophagy and protected dopaminergic neurons [9] and also upregulated Lc3-II levels and ameliorated pathology in a zebrafish model of tauopathy [10]. In mice, *Atg5* overexpression also increases autophagy [11]. Two candidates were selected as candidates to downregulate autophagy; mutant forms of *atg4b* and *bcl2l11*/*bim*. In yeast, the cysteine protease Atg4 is essential for cleaving the C terminus of Atg8, which is required for subsequent Atg8 – phosphatidylethanolamine (PE) conjugation [12]. In mammalian cells, expression of an inactive form of ATG4B with mutation in the catalytic domain (ATG4B^C74A^) inhibits LC3 lipidation, preventing closure of autophagic structures and impairing autophagic degradation [13]. BCL2L11 (BCL2 like 11) plays an important role in both apoptosis induction and autophagy inhibition [14]. Mutation of L152 and F159 in the BH3 domain of BCL2L11 (BCL2L11[EE]/BIM_EE), results in a protein which cannot bind to BCL2 (and hence does not affect apoptosis) but is still able to bind to BECN1 (beclin 1). Overexpression of BCL2L11[EE] in cultured cells results in downregulation of autophagy with no effect on cell death [15].

Using this inducible expression system, we have tested which transgenes robustly up- or downregulate autophagy and have validated these tools using Lc3-II blots, puncta analysis and disease rescue in a zebrafish model of neurodegeneration.

## Results

### Inducible expression of autophagy-related genes alters autophagy in zebrafish

**Figure 1.**
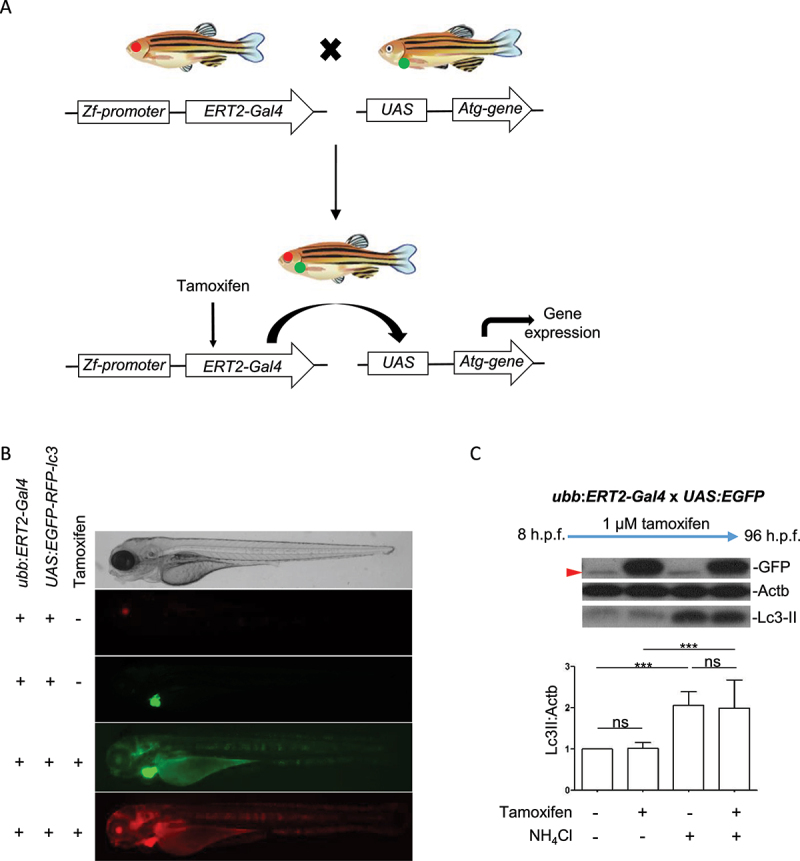
Regulation of transgene expression in *ERT2-Gal4* and *UAS* transgenic lines using tamoxifen. (a) Schematic diagram of crosses from *ERT2-Gal4* and *UAS* transgenic zebrafish. The *ERT2:Gal4* transgenes carry a *cryaa:RFP* cassette therefore transgenic fish can be identified by red fluorescence in the lens. The UAS transgenes carry a *myl7:EGFP* cassette which drives in GFP expression in the heart. This allows identification of transgenic carriers regardless of UAS expression. From crosses of *UAS* and *Gal4* transgenic fish, 25% of offspring will inherit both transgenes. (b) Tamoxifen induces *UAS*-driven transgene expression in double transgenic larvae. Offspring of *ubb:ERT2-Gal4* and *UAS:mRFP-GFP-lc3* were either treated with DMSO or 1 µM tamoxifen. In control (DMSO) conditions, ERT2-Gal4 is inactive as it is retained in the cytoplasm. Therefore, there is no *UAS*-driven expression in double transgenic zebrafish (red fluorescence in the lens and green fluorescence in the heart demonstrates that the larva carries both transgenes). Upon tamoxifen treatment, ERT2-Gal4 translocates to the nucleus, binds to *UAS* and activates transgene expression and both green and red fluorescence are seen throughout the body. (c) Tamoxifen treatment does not affect autophagic flux. Lc3-II levels were measured in double transgenic larvae carrying *ubb:ERT2-Gal4* and *UAS:EGFP* transgenes. In control conditions, GFP is not expressed. Treatment with 1 µM tamoxifen induces strong GFP expression but has no effect on Lc3-II levels in either basal or NH_4_Cl-treated conditions. A nonspecific band (red arrowhead) is present in all lanes and runs just below the level of GFP. This band is obscured by the GFP-positive band in lanes 2 and 4.

Inducible expression was achieved by crossing *ubb:ERT2-Gal4* fish with transgenic lines harboring different *UAS*-driven transgenes (see Table 2) and treating offspring with tamoxifen (Figure 1a). The *ubb:ERT2-Gal4* transgene carries the *cryaa:RFP* selection marker, therefore offspring which carry the transgene can be identified by RFP expression in lenses. All *UAS*-driven transgenes were generated with the *myl7:EGFP* selection marker therefore transgene-positive larvae can be identified by GFP expression in the heart. Crosses of *ubb:ERT2-Gal4* fish to *UAS:mRFP-GFP-lc3* fish were used determine that there was not “leaky” expression in the absence of tamoxifen (Figure 1b). In agreement with the results reported by Gerety et al. (2013), we observed that treatment with 1 µM tamoxifen from 8 h.p.f. was sufficient to drive strong ubiquitous transgene expression (GFP and RFP fluorescence, Figure 1b) and no transgene expression was observed in DMSO treatment groups [7]. To quantify the induction of expression achieved with 1 µM tamoxifen, we crossed *ubb:ERT2-Gal4* with *UAS:GFP* fish and treated offspring with either DMSO or 1 µM tamoxifen from 8 h.p.f. to 96 h.p.f. Larvae treated with DMSO had no detectable GFP expression (as determined by western blot and fluorescent imaging) whereas those with treated with tamoxifen had strong GFP expression (Figure 1c). In addition, we confirmed that treatment with 1 µM tamoxifen from 8 h.p.f. to 96 h.p.f. resulted in robust expression of GFP but did not affect Lc3-II levels compared to DMSO treated controls, either in basal or NH_4_Cl-treated conditions (Figure 1c).

**Table 2.** Zebrafish lines.

Abbreviated name of fish line	Construct	ZFIN ID
*UAS:atg5*	pTol2_*UAS:atg5-polyA,_ myl7:EGFP*	cu13Tg
*UAS:FLAG-atg4b_C74A*	pTol2_*UAS:FLAG-atg4b_C74A-polyA, myl7:EGFP*	cu14Tg
*UAS:His-bcl2l11[EE]*	pTol2_*UAS:His-bcl2l11_L128E_F135E-polyA, myl7:EGFP*	cu15Tg
*ubb:ERT2-Gal4*	p.*ubb:ERT2-Gal4-VP16, cryaa:RFP*	cu16Tg
*elavl3:ERT2-Gal4*	p. *elavl3:ERT2-Gal4-VP16, cryaa:RFP*	Cu29Tg
*UAS:mRFP-GFP-lc3*	pTol2_*UAS:mRFP-GFP-lc3-polyA, myl7:EGFP*	Cu25Tg
*UAS:Dendra*	pTol2_*UAS:Dendra2-polyA, myl7:EGFP*	Cu30Tg
*rho:GFP-MAPT/tau*	Generated previously by our lab [19]	Cu7Tg
*UAS:EGFP*	Generated by Lewis lab	No ZFIN ID

The existing lines used and those newly generated in this publication have been assigned the above identities by the Zebrafish Information Network, ZFIN (http://zfin.org/action/profile/view/ZDB-LAB-101027-4).

For all subsequent experiments, all offspring were treated with tamoxifen at a range of concentrations (0.1–3.0 µM) from 6–8 h.p.f. to 72 or 96 h.p.f. All concentrations were found to induce expression of the Dendra2 fluorescent protein in offspring from crosses of *ubb:ERT2-Gal4 and UAS:Dendra* fish and did not cause any overt morphological defects or toxicity (Fig. S1A). These concentrations were also tested in wild-type fish and did not affect autophagic flux (Fig. S1B). To examine whether overexpression of Atg5, Atg4b_C74A and Bcl2l11[EE] were sufficient to up- or downregulate autophagy, we crossed the *UAS*-driven autophagy modulator lines with *ubb:ERT2-Gal4* lines. Embryos from these crosses were treated with 1 µM tamoxifen treatment from 6–8 h.p.f. to 96 h.p.f. with daily replenishment of embryo medium and tamoxifen. Four hours prior to tissue collection, half the larvae were treated with ammonium chloride. Ammonium chloride raises the lumenal pH of the lysosome and hence prevents the degradation of Lc3-II/autophagosomes, like bafilomycin A_1_ and chloroquine. As these agents essentially clamp Lc3-II degradation, changes in its levels in the presence of these compounds can thus be ascribed to altered formation (autophagosome biosynthesis), and is an approach that is widely used in tissue culture, which has been adopted in *in vivo* models too [16].

**Figure 2.**
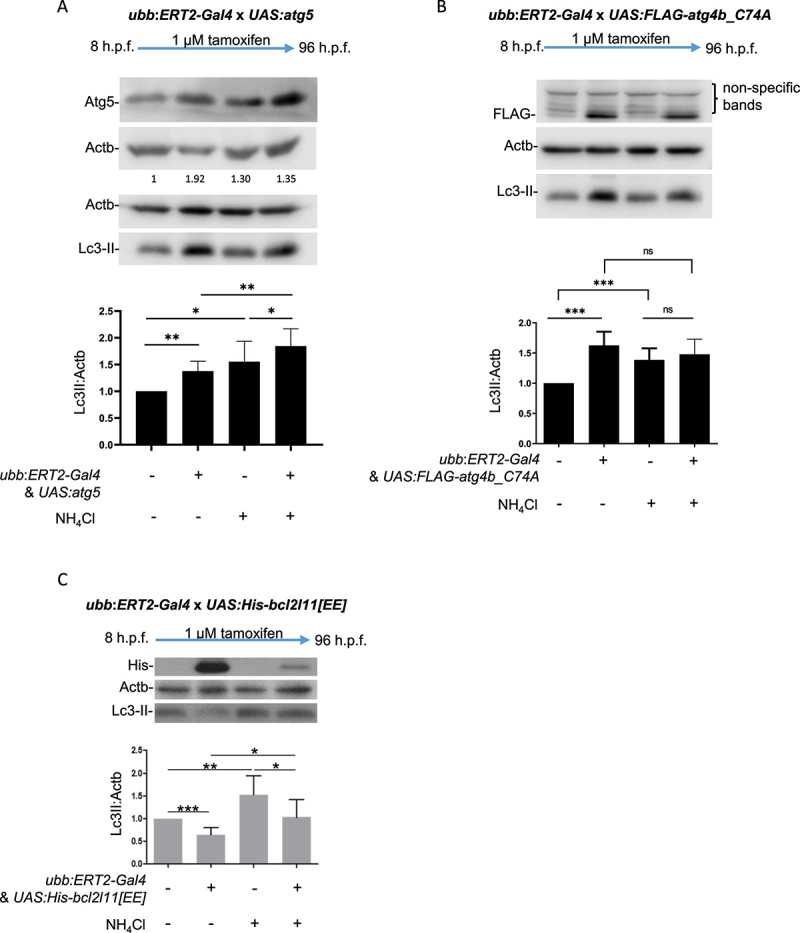
Regulation of transgene expression using *ERT2-Gal4* and *UAS* transgenic lines to control autophagic flux. (a) Induction of Atg5 expression results in upregulation of autophagy. All larvae from crosses of *ubb:ERT2-Gal4* and *UAS:atg5* were treated with 1 µM tamoxifen from 8 h.p.f. to 96 h.p.f. Endogenous Atg5 expression is detected in larvae which do not carry the transgenes. A significant increase in expression of Atg5 is observed in double transgenic larvae (resulting from *UAS*-transgene expression) and this correlates with increased Lc3-II. NH_4_Cl treatment is used to block lysosome acidification and therefore block autophagic flux. The increase in Lc3-II observed in Atg5 expressing larvae with NH_4_Cl treatment reflects the accumulation of autophagosomes which cannot be degraded. (b) Induction of Atg4b^C74A^ expression results in a block in autophagic flux. All larvae from crosses of *ubb:ERT2-Gal4* and *UAS:FLAG-atg4b_C74A* were treated with 1 µM tamoxifen from 8 h.p.f. to 96 h.p.f. Expression of the FLAG-tagged Atg4b^C74A^ transgene is observed in double transgenic larvae and this correlates with an increase Lc3-II. Lc3-II levels do not increase further in NH_4_Cl treatment conditions indicating that Atg4b^C74A^ expression causes a block in autophagic flux. Nonspecific bands were observed above the FLAG band in all treatment groups and genotypes. (c) Induction of Bcl2l11[EE] expression results in a downregulation of autophagy. All larvae from crosses of *ubb:ERT2-Gal4* and *UAS:His-bcl2l11[EE]* were treated with 1 µM tamoxifen from 8 h.p.f. to 96 h.p.f. Expression of His-tagged Bcl2l11[EE] is observed in double transgenic larvae and this correlates with a decrease in Lc3-II. Lc3-II levels increase in NH_4_Cl treatment conditions in both non-expressing and Bcl2l11[EE] expressing larvae as autophagosomes cannot be degraded. However, in Bcl2l11[EE] expressing larvae, Lc3-II levels remain significantly lower than non-transgenic siblings indicating a downregulation in autophagy. In all panels, graphs show mean values (± SEM) of densitometry of Lc3-II normalized to Actb (loading control) from >3 independent experiments. All graphs are normalized to the control (no transgene; no NH_4_Cl treatment) condition. Statistical analysis was performed using paired t-tests; ns – not significant; *p < 0.05; **p < 0.01; ***p < 0.001.

At the end of the treatment period, larvae were selected for the presence or absence of *Gal4* and *UAS* transgenes using *cryaa:mRFP* (red fluorescent lens) and *myl7:GFP* (green heart) selection markers for *Gal4* and *UAS* transgenes, respectively. Larvae that were positive for only a single transgene or neither transgene were used as controls. Endogenous Atg5 expression could be detected in all offspring with robust transgene expression detected in *Gal4/UAS*-positive offspring with tamoxifen treatment (Figure 2a). Atg5 overexpression resulted in an increase of Lc3-II levels compared to control (non-expressing) siblings and a further increase of Lc3-II levels was observed upon NH_4_Cl treatment, indicative of autophagy induction (Figure 2a). Overexpression of Atg4b^C74A^ was induced by tamoxifen treatment as detected by western blotting for the Flag-tag (Figure 2b). Lc3-II levels were increased in Atg4b^C74A^ expressing fish in basal conditions with no further increase observed when lysosome acidification was blocked with NH_4_Cl treatment, indicative of an autophagy flux-blocking effect (Figure 2b). Robust induction of Bcl2l11[EE] expression was observed in tamoxifen-treated transgenic larvae, as detected by western blotting for the His-tag. Bcl2l11[EE] overexpression resulted in a reduction of Lc3-II levels in basal conditions, indicative of a block in autophagosome synthesis and this reduction was also seen upon NH_4_Cl treatment, confirming the autophagy-blocking effect (Figure 2c). Although transgene-induced Atg5 expression does not appear to be as strong as that of Atg4b^C74A^ or Bcl2l11[EE], this likely reflects the affinity of the antibodies used. Atg5 was detected using an antibody against a synthetic peptide matched to the human and mouse protein sequence whereas Atg4b^C74A^ and Bcl2l11[EE] transgenes were tagged with Flag and His tags respectively. These antibody tags have the exact peptide sequence as that to which the antibody was raised and will therefore have greater affinity for the expressed protein and hence may appear to be expressed more strongly.

We next examined the range of concentrations of tamoxifen able to induce robust expression of the transgenes. Atg4b^C74A^ expression was strongly induced by 0.5 µM tamoxifen from 8 h.p.f to 96 h.p.f. and caused a significant block in autophagy flux (measured by Lc3-II levels, Figure S2A), whereas no obvious Atg4b^C74A^ expression and an inconsistent effect on Lc3-II were observed with 0.1 µM tamoxifen treatment for the same exposure duration (Figure S2B). Similarly, the use of 0.5 µM tamoxifen resulted in strong Atg5 expression and a significant increase in Lc3-II levels in basal and ammonium chloride treatment conditions, indicating an increase in autophagic flux (Figure S2c), whereas 0.1 µM tamoxifen treatment did not induce Atg5 expression (Figure S2d,e) and had no effect on Lc3-II levels in either basal or ammonium chloride treatment conditions (Figure S2d,f).

**Figure 3.**
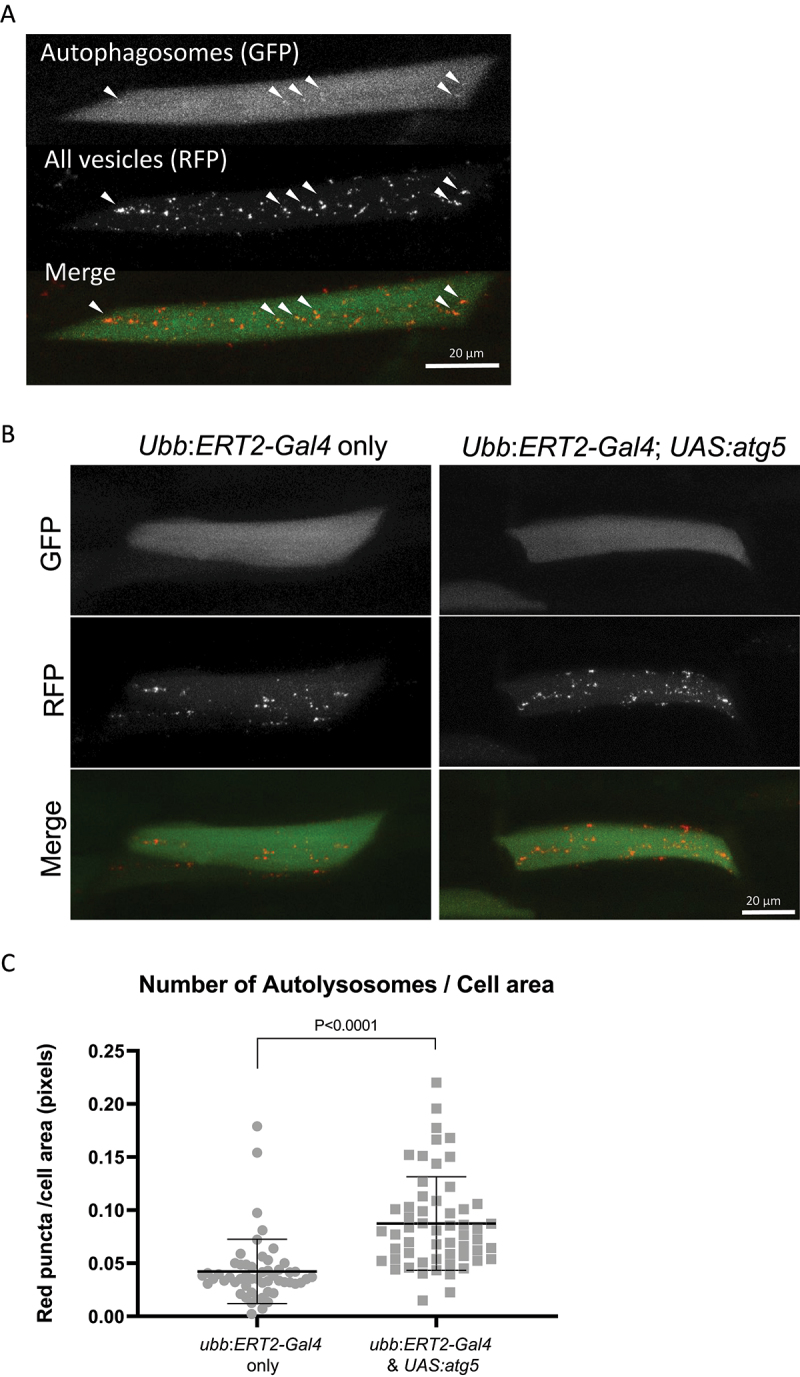
Expression of Atg5 increases autophagic flux in muscle cells. Eggs from a *ubb:ERT2-Gal4* x *UAS:atg5* cross were injected with the *UAS:mRFP-GFP-lc3* reporter and treated with 1 µM tamoxifen from 8 h.p.f. The numbers of autophagosomes and autolysosomes were quantified in individual muscle cells of larvae at 96 h.p.f. (a) Representative images of a muscle cell expressing mRFP-GFP-lc3, a dual-fluorescent reporter for the quantification of autophagic flux. Autophagosomes are visualized as bright puncta evident in both green and red channels (white arrowheads). Red puncta without any green signal correspond to autolysosomes, since the GFP signal has been quenched by the low pH following lysosome fusion. Very few autophagosomes (yellow puncta) are observed compared to autolysosomes (red only puncta) with this reporter construct suggesting autophagosomes rapidly fuse with the lysosome. Images shown are the maximum intensity projections of the green and red channel z-stacks. Scale bar: 20 μm. (b) Representative images of muscle cells expressing mRFP-GFP-lc3 in larvae overexpressing Atg5 and their corresponding non-expressing siblings from a cross *of ubb:ERT2-Gal4* x *UAS:atg5* fish. Red vesicles (autolysosomes) were more abundant in Atg5-expressing larvae. Few cells were found with yellow vesicles (autophagosomes) in either experimental group. Scale bar: 20 μm. (c) Quantification of autolysosomes per cell area calculated from maximum intensity projection of the red channel of individual muscle cells. Overexpression of Atg5 resulted in a statistically significant increase in autolysosomes. Graph represents the numbers of red puncta normalized to the area of the cell (in pixels) (N = 53 control muscle cells; N = 57 atg5-expressing muscle cells; P < 0.0001).

In addition to western blot analysis, we determined the number of autophagosomes and autolysosomes as a quantitative readout of autophagic flux in zebrafish. The tandem fluorescent mRFP-GFP-LC3 probe has been widely used in cell culture and also *in vivo* to measure the number of autophagosomes and autolysosomes [17]. Lc3 associates with pre-autophagosomal structures, mature autophagosomes and also autolysosomes. The *mRFP-GFP-lc3* transgene can be used to label and therefore count the number of vesicles and to discriminate between autophagosomes and autolysosomes, since the GFP signal becomes quenched in the acidic lysosomal environment. We used DNA injections of *UAS:mRFP-GFP-lc3* into offspring of crosses of *ubb:ERT2-Gal4* with *UAS:atg5* fish. DNA injections result in mosaic expression and therefore, when expression is induced by tamoxifen, allows the analysis of expression in individual cells (n.b. tamoxifen treatment induces the expression of both *UAS:mRFP-GFP-lc3* and *UAS:atg5*). Confocal images of the red and green signal of individual muscle cells were used to analyze the green and red puncta. Autophagosomes were visualized as green puncta colocalizing with red puncta whereas autolysosomes were identified as red only puncta (Figure 3a). This analysis allows the quantification of the number of autophagic vesicles, and can be used to identify changes in the autophagic flux. A significant increase in RFP-only positive vesicles was observed in larvae when Atg5 was overexpressed compared to *ubb:ERT2-Gal4* only siblings (Figure 3b,c), indicating an increase in the numbers of autolysosomes. The area of each individual red puncta was measured in every cell using ImageJ. A total of 2838 vesicles were counted in the control group (n = 53 cells; average of 54 vesicles per cell) versus a total of 5988 vesicles in Atg5 overexpressing larvae (n = 57 cells; average of 105 vesicles per cell), corroborating the increase in autophagic vesicles counted manually. The size of autophagic vesicles was not influenced by Atg5-induced autophagy induction (Fig. S3A). The majority of puncta observed were red-only (Figure 3a,b) supporting the idea that autophagosomes rapidly fused with lysosomes. However, the proportion of cells containing autophagosomes was greater in Atg5 overexpressing larvae compared to their respective controls, indicating that Atg5 expression does increase the abundance of autophagosomes (Fig. S3B). Despite this, no significant changes were found in the total numbers of autophagosomes per cell between the two experimental groups (Fig S3C).

## Temporal and spatial control of autophagy-related gene expression

**Figure 4.**
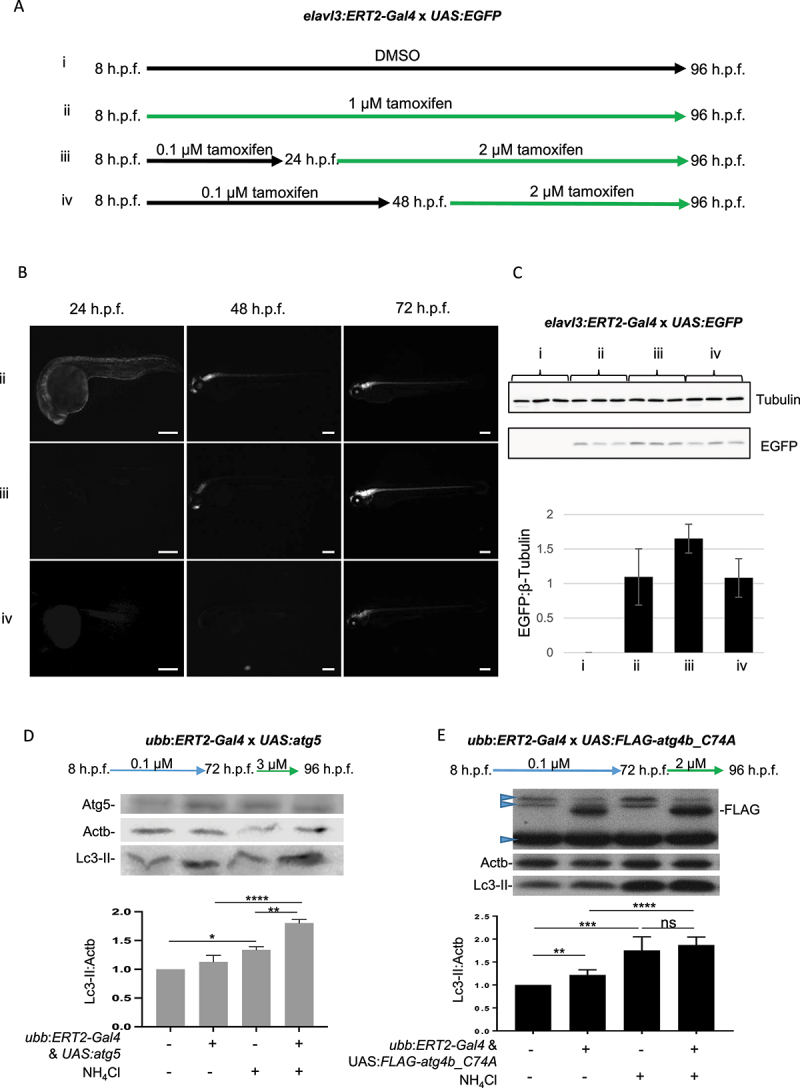
Temporal control of transgene expression to regulate autophagic flux. (a-c) Larvae from crosses of *elavl3:ERT2-Gal4* and *UAS:EGFP* were used to optimize the timing and concentration of tamoxifen treatment for temporal control of transgene expression. (a) Schematic diagram of different treatment conditions. (b) Treatment of larvae from 8 h.p.f. to 96 h.p.f. with 1 µM tamoxifen (condition ii) resulted in transgene expression which was evident from 24 h.p.f. and strongly expressed in the CNS from 48 h.p.f. onwards. Representative images taken using GFP filter (excitation 395–455 nm; emission 480 nm). (b and c) Treatment with 2 µM tamoxifen was required to induce GFP expression at later time points, from 24 or 48 h.p.f. onwards (conditions iii and iv, respectively). (c) Western blot and quantification of EGFP protein levels (samples run from 3 independent experiments on single gel). (d) Temporal induction of Atg5 expression results in upregulation of autophagy. All larvae from crosses of *ubb:ERT2-Gal4* and *UAS:atg5* were treated with 0.1 µM tamoxifen from 8 h.p.f. to 72 h.p.f. to prime transgene expression and then with 3 µM tamoxifen from 72 h.p.f. to 96 h.p.f. Atg5 expression was induced, although not as strongly as when treatment was initiated early (quantified in Fig. S1B). Although no increase in Lc3-II was observed in basal conditions, the strong increase in Lc3-II observed in Atg5 expressing larvae with NH_4_Cl treatment, which is much greater than that observed in non-transgenic siblings with NH_4_Cl treatment, demonstrates that late induction was able to induce autophagic flux. (e) Temporal induction of Atg4b^C74A^ expression results in a block in autophagic flux. All larvae from crosses of *ubb:ERT2-Gal4* and *UAS:FLAG-atg4b_C74A* were treated with 0.1 µM tamoxifen from 8 h.p.f. to 72 h.p.f. to prime transgene expression and then with 2 µM tamoxifen from 72 h.p.f. to 96 h.p.f. Strong induction of FLAG-tagged Atg4b^C74A^ was observed in double transgenic larvae with this late induction protocol which correlated with an increase Lc3-II expression. Lc3-II levels did not increase in controls versus Atg4b^C47A^ expressing siblings in NH_4_Cl treatment conditions indicating that Atg4b^C74A^ expression causes a block in autophagic flux. Nonspecific bands (blue arrowheads) were observed in all treatment groups and genotypes. (d and e). Graphs show mean values (± SEM) of densitometry of Lc3-II normalized to Actb (loading control) from at least 3 independent experiments. All graphs are normalized to the control (no treatment) condition. Statistical analysis was performed using paired t-tests: ns – not significant; *p < 0.05; **p < 0.01; ***p < 0.001, ****p < 0.0001.

To investigate tissue-specific control of transgene expression, we used the *elavl3:ERT2-Gal4* driver line and investigated the strength and localization of GFP expression compared to *Ubb:ERT2-Gal4* driven expression. Treatment with 1 µM tamoxifen from 8 h.p.f. resulted in robust, ubiquitous transgene expression at 24 h.p.f. and this remained strong at 48 h.p.f. In larvae from crosses using the *elavl3:ERT2-Gal4* driver treated with 1 µM tamoxifen, transgene expression was observed at 24 h.p.f. in the developing nervous system and became more evident and localized to the CNS 48 h.p.f. (Fig. S4A). We next tested whether this system could be used for temporal control of transgene expression, by altering the timepoints of tamoxifen treatment. We found that adding tamoxifen at 1, 2 or 3 µM from 24 h.p.f. and later time points did not induce expression (data not shown). We hypothesized that low concentrations of tamoxifen might be used to “prime” transgene expression, followed by later higher tamoxifen exposure to drive strong overexpression. We previously observed that 0.1 µM tamoxifen induced weak expression in *Ubb:ERT2-Gal4; UAS:Dendra2* fish (Fig. S1A) but did not induce transgene expression in *UAS*-driven autophagy lines (Figure 4b,d-f). We used this concentration from 8 h.p.f., followed by a later, higher concentration of tamoxifen to optimize late transgene induction. We found that treatment of *elavl3:ERT2-Gal4* x *UAS:EGFP* fish with 0.1 µM tamoxifen from 8–24 h.p.f., then 1 µM tamoxifen for a further 72 h did not induce strong EGFP expression (data not shown). However, treatment with 0.1 µM tamoxifen from 8–24 h.p.f., then with 2 µM tamoxifen from 24 or 48 h.p.f. onwards resulted in robust GFP expression (Figure 4a-c), localized only to the CNS. Similarly, we found that treatment of *Ubb:ERT2-Gal4* x *UAS:atg5* or *UAS:FLAG-atg4b_C74A* larvae with 0.1 µM tamoxifen from 6–72 h.p.f., then 1 µM tamoxifen for 24 h did not induce strong enough transgene expression to alter autophagic flux (data not shown). However, treatment of *Ubb:ERT2-Gal4 x UAS:atg5* larvae with 0.1 µM tamoxifen from 8–72 h.p.f., then 3 µM tamoxifen for 24 h resulted in induction of Atg5 expression (Fig S4B). Although this resulted in only a modest increase in Lc3-II levels in basal conditions, a significant increase in Lc3-II was observed in NH_4_Cl conditions, demonstrating that autophagy was induced in older larvae with this protocol (Figure 4d). Using a similar protocol in *Ubb:ERT2-Gal4* x *UAS:FLAG-atg4b_C74A* larvae, treatment with 0.1 µM tamoxifen from 8–72 h.p.f., then 2 µM tamoxifen for 24 h resulted in robust Atg4b^C74A^ transgene expression, with an increase in Lc3-II levels observed in Atg4b^C74A^ expressing larvae in basal conditions and no difference between control and Atg4b^C74A^ expressing larvae in NH_4_Cl treatment, indicating a block in autophagic flux (Figure 4e).

## Autophagy upregulation by induction of Atg5 expression ameliorates tau pathology in the zebrafish retina

**Figure 5.**
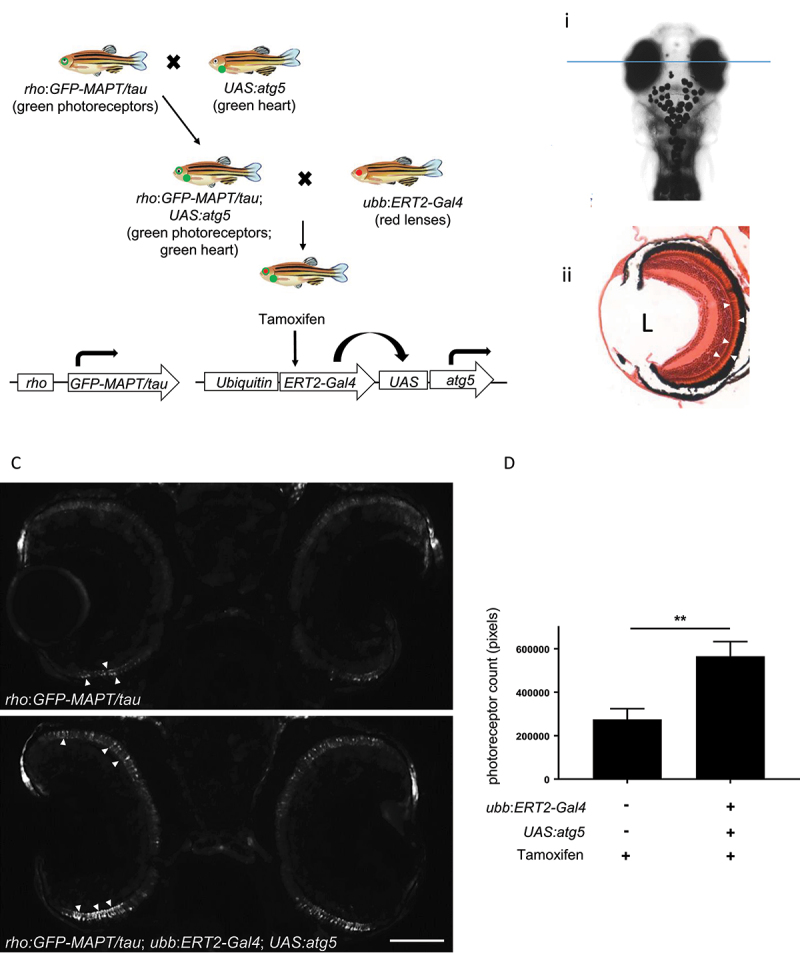
Autophagy upregulation by induction of Atg5 expression ameliorates pathology in a zebrafish model of tauopathy. (a) Schematic diagram of crosses to generate triple transgenic zebrafish. Photoreceptor degeneration is observed in the zebrafish transgenic line expressing the GFP-tagged human *MAPT* gene under the control of the rhodopsin promoter (*rho:GFP-MAPT/tau* transgene). This degeneration has previously been shown to be ameliorated by pharmacological upregulation of autophagy [19]. The *rho:GFP-MAPT/tau* line was crossed to the *UAS:atg5* transgenic line and double transgenic fish were identified as those with GFP expression in the retina and in the heart. These double transgenic fish were crossed with *ubb:ERT2-Gal4* transgenic fish. From the final cross, 12.5% of offspring will inherit all 3 transgenes (GFP in retina and heart; RFP in the lens). All offspring were treated with 1 µM tamoxifen from 8 h.p.f. to 9 d.p.f. At the end of the treatment period, larvae were sorted for expression by GFP and RFP expression to identify those expressing only the *rho:GFP-MAPT/tau* transgene and those expressing all three transgenes. (b) Photoreceptor degeneration analysis. (I) Cryosections through the central retina (plane of section shown by blue line) were imaged and quantified to determine the photoreceptor number and distribution. (ii) Hematoxylin and eosin-stained histological section to demonstrate the photoreceptor layer (arrowheads). L marks position of lens. (c and d) By 9 d.p.f., almost all photoreceptors have degenerated in *rho:GFP-MAPT/tau fish* (upper panel) with just a few GFP-positive cells (arrowheads) remaining at the ciliary marginal zone. In triple transgenic zebrafish (overexpressing Atg5), there is a significant rescue of photoreceptor degeneration. Representative images taken using GFP filter (excitation 395–455 nm; emission 480 nm). Quantification of photoreceptors is shown in (d), (n ≥ 55 eyes analyzed per group; unpaired t-test, **p < 0.01). Scale bar: 50 µm.

We and others have previously shown that small changes in autophagic flux, as measured by changes in Lc3-II and Lc3-positive puncta, can have a marked effect on the clearance of autophagy substrates [18], hence this may be a more physiologically relevant method to measure the impact of altering autophagic flux. To investigate whether modulation of autophagy through our inducible system was sufficient to affect disease pathology caused by the accumulation of an aggregate-prone autophagy substrate, we crossed the *UAS:atg5* transgenic line to a zebrafish model of tauopathy. In this model, GFP-tagged human wild-type MAPT/tau is expressed in the rod photoreceptors in the retina and results in increasing photoreceptor degeneration over time [19]. The *rho:GFP-MAPT/tau; UAS:atg5* double transgenic fish were crossed to *ubb*:ERT2-Gal4 transgenic fish (Figure 5a) and all offspring treated with 1 µM tamoxifen from 8 h.p.f. to 9 d.p.f. Photoreceptor degeneration was assessed by quantifying the number of GFP-positive photoreceptors present in sections through the central retina (Figure 5b,c). Striking degeneration was observed in *rho:GFP-MAPT/tau* fish which were negative for *Gal4* and *UAS* transgenes, whereas significant rescue of photoreceptors was observed in offspring expressing Atg5 (Figure 5d). This effect was confirmed to be caused by the expression overexpression of Atg5, since tamoxifen treatment in *rho:GFP-MAPT/tau* fish (i.e. without the *UAS:atg5* transgene) had no effect (Fig. S5).

## Discussion

Until recently, one of the limitations of using zebrafish to study biological processes or disease progression was the lack of tools to temporally control and manipulate gene expression. Transient overexpression and knock-down can be achieved by injections of *in vitro* synthesized mRNA for overexpression of a specific protein, or with morpholino oligonucleotides (MOs) for reducing translation, respectively [20]. Overexpression can also be achieved by injection of DNA constructs, although this usually results in mosaic expression [21]. However, with such approaches, there are significant limitations to the use of these techniques to control gene expression. Constructs are typically injected into the 1 cell stage embryo and results in transient alterations in gene expression, depending on the speed of mRNA or DNA degradation and the rate at which the MO is bound to local mRNA. Furthermore, since embryos are injected at the one cell stage, the injected mRNA, DNA or MO is transmitted to all cells during development, hence there is no spatial or temporal control of knockdown or overexpression; after delivery, the knockdown or overexpression nucleotide is permanently “on”. If protein overexpression or knockdown at early embryonic stages causes lethality or early developmental defects, this precludes any observations on later larval stages. Delivery of either overexpression constructs or MOs can be achieved at later larval stages via electroporation which give localized but not tissue-specific delivery and again, is limited by the degradation rate of the overexpressed or knockdown nucleotides.

As an improved alternative method to these transient approaches, an inducible system for control of transgene expression has been developed in zebrafish [7,22]. In addition to demonstrating temporal expression of fluorescent proteins (e.g. for cell lineage tracing studies), they further showed that the inducible system was able to drive gain-of-function expression using a dominant-negative caspase9 construct to block drug-induced apoptosis [7] or to induce tissue-specific Notch activation [22]. This technology has since been used for temporal control of *ascl1b* expression to confirm the biological target responsible for valproic acid-induced changes in 5-HT neuronal differentiation [23] and of *apoa1bp2* (Aibp2) to investigate cholesterol synthesis [24]. In addition, several studies have used tissue-specific ERT2-Gal4 expression to achieve spatial and temporal control: for example, constitutively active Rho expression and EphA4a expression to investigate the role actinomyosin cables in embryonic hindbrain boundary formation [25]; to drive vSrc and dominant-negative Rab5 expression in epithelial cells to investigate their apical elimination following transformation [26]; and to drive expression of nuclear-localized Srebp2 in an Aibp2-deficient background to rescue haematopoetic stem cells (HSC) and thereby confirm that that Srebp2 acts downstream of Aibp2 to orchestrate HSC specification [24]. In addition, spatial control can be achieved by using “caged” tamoxifen analogues which are only active upon photoactivation [27–29].

One potential drawback of using the *ERT2-Gal4/UAS* transgene system is that some *UAS*-driven transgenes have been reported to become silenced in successive generations. Goll *et al*. demonstrated that the UAS binding site contains CpG motifs that can become methylated and the presence of this methylation is thought to prevent Gal4 binding and therefore lead to silencing [30]. They found that Gal4 binding to the UAS sites was sufficient to prevent methylation and this may provide an explanation for the need to “prime” with a low concentration of tamoxifen to achieve later expression of the *UAS*-driven transgenes. However, we have maintained the *UAS*-driven transgenic lines reported here for at least four generations without silencing.

Here, we have validated a suite of tools for temporal autophagy up- and downregulation *in vivo*. Our work is particularly focussed on the role of autophagy in neurodegeneration and we have demonstrated the utility of these tools for manipulating autophagy in the context of neurodegenerative disease. In future studies, this technology will allow us to manipulate autophagy in particular cell types. Drivers for tissue-specific expression of *GAL4-ERT2* have already been described for neurons (*HuC:ERT2-Gal4* [7]), epithelial cells (*krt18a.1:KALTA4-ERT2* and *krt5:Gal4-ERT-VP16* [22,26],) and endothelial cells (*kdrl:Gal4-ERT2* [24]) and others can be easily generated using with cloning techniques such as Gateway recombination and Gibson assembly [31,32]. In addition, our autophagy regulation system can also be used in conjunction with conventional *Gal4* driver lines, of which 3,759 fish stocks are currently listed containing *Gal4* transgenes on the ZFIN database (https://zfin.org/). Although there are numerous duplicate entries of the same *Gal4* lines (for example, on different transgenic or mutant backgrounds), this is likely an underestimation of the number of available lines, given the output of enhancer trap screens, such as zTrap [33] and tissue-specific screens [34,35]. Additionally, temporal control could be achieved without generating *ERT2-Gal4* lines by using *Hsp70:Gal4*, with temporal control by immersion in 39°C embryo medium buffer [36] or localized expression using laser warming [37,38].

We believe these tools will be useful across a wide range of fields including development, cancer biology, inflammation and liver disease, for which tissue-specific *Gal4* driver lines already exist. In addition, the recent developments demonstrating CRISPR-Cas9 knock-in of transgenes at an existing transgene locus (e.g. GFP reporter) or to endogenous genes, suggests that it would be similarly possible to knock-in *Gal4-ERT2* transgenes [39,40], which would further increase the utility of this technology.

## Materials and methods

### Maintenance of stocks and collection of embryos

All zebrafish procedures were performed in accordance with the UK Animals (Scientific Procedures) Act with appropriate Home Office Project and Personal animal licenses and with local Ethics Committee approval. Studies were performed in accordance with PREPARE and ARRIVE guidelines. Zebrafish were kept and grown up on a 14-h light:10-h dark cycle under standard conditions. Embryos were collected from natural spawnings, staged according to established criteria [41] and reared in embryo medium (5 mM NaCl, 0.17 mM KCl, 0.33 mMCaCl_2_, 0.33 mM Mg_2_SO_4_, 5 mM HEPES, pH 7.2) at 28.5°C in the dark.

### Generation of transgenic constructs

Transgenic constructs were generated via Gateway recombination using components of the zebrafish Tol2kit [42]. A zebrafish *atg5* cDNA clone (Source Bioscience, MGC 100934, IMAGE:7145909) served as a template for add-on PCR of a 5’ attL1 site and Kozak sequence and 3’ attL2 site making the product compatible with Multisite Gateway® cloning technology (ThermoFisher). Recombination of the PCR product and the pDONR221 vector clone was performed according to the manufacturer’s instructions, thereby creating the pME_atg5 middle entry clone. Sequence alignment and analysis was performed to identify the equivalent amino acids in zebrafish *Bcl2l11* and *Atg4b* to those in the mammalian sequence for mutagenesis. Zebrafish Bcl2l11^L128E^ is equivalent to mammalian L152E and zebrafish Bcl2l11^L135E^ is equivalent to mammalian F159E; residues in Atg4b are at the same position in the zebrafish and mammalian peptide sequence. For creation of pME_*FLAG-atg4b_C74A* and pME_*His-bcl2l11_L128E_F135E* constructs, RNA was isolated from 5 days post-fertilization (d.p.f.) wild-type larvae using RNeasy mini kit (Qiagen, 74,134). Following DNase treatment (Invitrogen, 18,068,015), 1 µg of RNA was reverse transcribed using the High Capacity cDNA reverse transcription kit (Applied Biosystems, 4,368,814). *Atg4b* and *Bcl2l11* cDNA were amplified by PCR and the products cloned into pCR-TOPO using the TOPO TA cloning kit (ThermoFisher, K460001) following the manufacturer’s instructions. The resulting pCRII_*atg4b* plasmid was used for site-directed mutagenesis creating *atg4b_C74A* followed by add on PCR of a 5’ attL1 site, Kozak sequence and FLAG-tag and a 3’ attL2 site on *atg4b_C74A* using a M13F site for primer binding. TOPO cloning-derived pCRII_*Bcl2l11* was used as a template for two-step site-directed mutagenesis. Sequence analysis identified the L128 and F135 sites in zebrafish Bcl2l11, to be equivalent to L152 and F159 in mammalian BCL2L11. Two-step site-directed mutagenesis was performed to generate Bcl2l11^L128E,F135E^ (hereafter called Bcl2l11[EE]) followed by add on PCR of a 5’ attL1 site, Kozak sequence, His-tag and a 3’ attL2 site using M13F. All PCR products were processed as described above for *atg5*, generating pME_*FLAG-atg4b_C74A* and pME_*His-bcl2l11[EE]* clones. In pilot experiments using both wild-type zebrafish and embryos injected with zebrafish cDNA constructs, zebrafish Atg5 protein could be detected using antibodies raised against a synthetic peptide of ATG5 (Abcam, ab108327) whereas Bcl2l11[EE] and Atg4b proteins could not be detected with commercially available antibodies (raised against mouse or human forms of these proteins), hence the *bcl2l11[EE]* and *atg4b_C74A* constructs were tagged with His and Flag tags, respectively. *mRFP-GFP-LC3* (a gift from T. Yoshimori, Osaka University, Japan) was excised from the original plasmid (described in [43]) using NheI and SacII and cloned into the pME-MCS vector [42] at SpeI and SacII sites. Zebrafish *map1lc3b* (hereafter called *lc3*) was amplified from cDNA using sequence-specific primers with additional BspeI and SacI sites and these sites were used to replace rat *LC3* in the pME-*mRFP-GFP-LC3* construct to create pME-*mRFP-GFP-lc3. Dendra2* was excised from pDendra2 plasmid (Evrogen, FP821) and subcloned into the pME-MCS vector at Nhe1 and Sac1 sites to create pME-*Dendra2*. All primers used in construct generation are provided in Table S1.

The transgenic constructs pTol2 _UAS*:atg5-polyA*, pTol2 _UAS*:FLAG-atg4b_C74A-polyA*, pTol2 _UAS*: His-bcl2l11[EE]-polyA*, pTol2_*UAS:mRFP-GFP-lc3-polyA* and pTol2_*UAS:Dendra2-polyA* were generated via Gateway recombination using the pME vectors described above together with p5E-*UAS* and p3E-*polyA* components of the Tol2 kit within a destination vector (pDestTol2CG2) [42] according to the manufacturer’s procedures. All of the above transgenic constructs contain reporter comprising the myosin light chain 7 promoter driving EGFP (*myl7:EGFP*) as a selection marker to allow the identification of UAS-transgene-positive offspring. The ubiquitin enhancer construct driving *ERT2-Gal4-VP16* (*ubb:ERT2-Gal4-VP16*) and the neuronal (HuC) enhancer/promoter construct driving *ERT2-Gal4-VP16* (*elavl3*:ERT2-Gal4-VP16) containing a miniTol2 backbone and an *alpha-crystallin:RFP* (*cryaa:RFP*) cassette as selection marker were kindly provided by Sebastian S. Gerety (Wellcome Sanger Institute, UK) [7]. The *UAS:EGFP* line was a gift from Kate Lewis (Syracuse University, NY), generated using components of the Tol2kit by Gustavo Cerda (University of Cambridge, UK). A list of the transgenic constructs generated is provided in Table 1.

**Table 1.** Injected plasmids.

pTol2_*UAS:atg5-polyA, myl7:EGFP*	Generated in this study
pTol2_*UAS:FLAG-atg4b_C74A-polyA, myl7:EGFP*	Generated in this study
pTol2_*UAS:His-Bcl2l11_L128E_F135E-polyA, myl7:EGFP*	Generated in this study
pTol2_*UAS:mRFP-GFP-lc3-polyA, myl7:EGFP*	Generated in this study
pTol2_*UAS:Dendra2-polyA, myl7:EGFP*	Generated in this study
p.*ubb:ERT2-Gal4-VP16, cryaa:RFP*	Gift from S. Gerety
p. *elavl3:ERT2-Gal4-VP16, cryaa:RFP*	Gift from S. Gerety

### Plasmid injections and generation of transgenic fish

On the day of injection, the DNA constructs and Tol2 transposase mRNA were prepared at a final concentration of 80 ng/µl of DNA and 25 ng/µl of RNA in Danieau’s solution (58 mM NaCl, 0.7 mM KCl, 0.4 mM MgSO_4_.7H_2_O, 0.6 mM Ca[NO_3_]_2_, 5 mM HEPES, pH 7.6) containing 20% phenol red as previously described [10]. The DNA/mRNA mixture was injected into the cell of 1 cell-stage embryos of wild-type zebrafish. At 3 d.p.f., embryos were selected based on the expression of selection markers (*cryaa:RFP* results in red fluorescent protein expression in the lenses in the *ERT2-Gal4* lines and *myl7:EGFP* results in green fluorescent hearts in the *UAS* lines). Embryos with positive signal, observed using mRFP and EGFP filter sets on an Olympus SZX12 stereo fluorescence microscope, were raised to adulthood. Adult G0 were outcrossed to TL or AB wild-type fish and the F0 generation was screened to identify embryos with green hearts or red lenses. These embryos were raised to establish transgenic heterozygous lines which have subsequently been bred for at least 3 generations. A list of the zebrafish lines generated in this study is provided in Table 2.

### Experimental crosses

Offspring of crosses of *ubb:ERT2-Gal4* or *elavl3:ERT2-Gal4* with *UAS:EGFP, UAS:Dendra2* or *UAS:mRFP-GFP-lc3* were used to optimize tamoxifen treatments. Embryos and larvae for autophagy regulation experiments were generated from crosses of *ubb:ERT2-Gal4* fish with each of the *UAS*-driven autophagy modulator lines (*ubb:ERT2-Gal4* x *UAS:FLAG-atg4b_C74A, ubb:ERT2-Gal4* x *UAS:atg5, ubb:ERT2-Gal4* x *UAS:His-bcl2l11[EE]*). From experimental crosses, fish were screened for GFP expression in hearts (selection marker for *UAS*-lines) and RFP expression in lenses (selection marker for *ubb*:ERT2-Gal4 and *elavl3:ERT2-Gal4* positive fish) and selected and sorted into groups for subsequent analysis (negative for one or both transgenes *vs* both GFP positive and RFP positive). To image and analyze autophagic vesicles upon Atg5 overexpression, offspring from *ubb:ERT2-Gal4* x *UAS:atg5* cross were injected with 40 ng/µL of pTol2_*UAS:mRFP-GFP-lc3-polyA* DNA and 25 ng/µl of transposase RNA in Danieau’s solution containing 20% phenol red. Larvae were reared in EM and treated with 0.003% phenylthiourea from 24 h post-fertilization (h.p.f.) to prevent pigmentation. Activation of the Gal4-UAS system was induced by the addition of 0.1–3 μM tamoxifen at 6 h.p.f. – 8 h.p.f. with daily replenishment of EM and tamoxifen at same concentration until 96 h.p.f., prior collection for imaging. For puncta analysis, larvae with mosaic fluorescent expression of the *mRFP-GFP-lc3* reporter were screened and selected under a fluorescent microscope with EGFP and RFP filters. All larvae with mosaic expression of the *mRFP-GFP-lc3* construct were positive for GAL4 (red lenses). Atg5 overexpressing larvae were identified by their bright and homogeneous fluorescent green heart, whereas controls (with no Atg5 expression but with mRFP-GFP-lc3 signal) were distinguished by mosaic cardiac GFP expression resulting from *mRFP-GFP-lc3* reporter DNA injection. At 96 h.p.f., larvae were anaesthetized by immersion in 0.2 mg/ml 3-amino benzoic acid ethylester (MS222, also known as tricaine), fixed in 4% PFA for 2 h at room temperature and washed in PBS. Larvae were kept in PBS at 4°C and were imaged within 24 h of fixation.

### Confocal microscopy

Analysis of mRFP-EGFP-lc3 puncta was performed on fixed transgenic larvae at 96 h.p.f. mounted in 0.75% low melting point agarose in embryo medium in a confocal imaging chamber. Muscle cells were imaged using a Leica SP8 confocal with a 40x oil-immersion objective and an optical magnification of x2 using the 488 nm excitation laser to image the GFP signal and 552 nm excitation laser for RFP. Green and red emission signals were detected and collected in the ranges of 491 nm-564 nm and 565 nm-750 nm, respectively, and imaged sequentially to avoid crosstalk. 1-micron-step z-stacks were taken of whole muscle cells. A minimum of 53 muscle cells in 25 larvae were imaged per group. Confocal images were analyzed using FIJI software (ImageJ). Each z-stack was converted to a maximum intensity projection image and the numbers of red and green puncta were counted manually. The area of the cell was measured by creating a region of interest (ROI) delimiting the cell shape and confirming the boundary in images from both channels. To analyze the size of the autophagic vesicles, maximum intensity projections were converted to binary images. The size of all vesicles within each cell was measured using the “analyze particles” tool in ImageJ.

### Western blotting

Western blots were performed as previously described [10] with minor modifications. 10 larvae were collected per sample and each experiment was performed in triplicate. Total protein for each lysate was determined using a BCA assay (Sigma). 30 µg of protein was loaded per lane. Immunoreactive bands were then detected using Enhanced Chemiluminescent substrate (GE Healthcare Bioscience) then either mounted in cassettes for exposure with Amersham Hyperfilm and processed using a Fuji FPM 100A and scanned using an A4 flatbed scanner for analysis on the computer or membranes were imaged using a LI-COR Odyssey-Fc, model 2800, and Image Studio Version 5.2 software. Densitometry was performed using Fiji. Background was subtracted from images and the image is inverted to obtain a black background and white bands. A rectangular region of interest (ROI) around the biggest band was drawn then copied to the other bands. Integrated Density was measured within the ROIs. The ratio of Integrated Density of the bands corresponding to a specific antibody and its respective loading control was used for the normalization. All antibodies were tested with a range of protein concentrations to ensure that experimental samples were analyzed within the linear detection range. The following antibodies were used: mouse anti-ACTB/β-actin antibody (Sigma), dilution 1:1000; mouse anti-His antibody (Qiagen), dilution 1:1000; rabbit anti-ATG5 antibody (Abcam), dilution at either 1:250 or 1:1000; rabbit anti-LC3B (Novus Biologicals), dilution 1:1000; rabbit anti-GFP antibody (Abcam) dilution 1:1000; mouse anti-FLAG (Sigma), dilution 1:1000. Further details of antibodies used in this study are provided in Table 3.

**Table 3.** Antibodies.

Mouse anti-ACTB/β-actin	Sigma, A5316
Mouse anti-RGS-His	Qiagen, 34,610
Rabbit anti-ATG5	Abcam, ab108327
Rabbit anti-LC3B	Novus Biologicals, NB100-2220 lot AV-1
Mouse anti-FLAG	Sigma, F-3165
Rabbit anti-GFP	Abcam, ab290-50

### Tamoxifen treatment

A 100 mM stock of 4-OH-tamoxifen (Sigma) in dimethyl sulfoxide (DMSO) was prepared and stored at −20°C. To induce gene expression in experimental crosses, varying concentrations of tamoxifen were added to the embryo medium at different timepoints, from 6–8 h.p.f. onwards. Embryos were left in their chorions until autonomous hatching as pilot experiments demonstrated that gene expression could be induced without chorion removal. Final concentrations used in this study ranged from 0.1 µM to a maximum of 3 µM. Initial treatment was followed by daily exchange of half of the volume of embryo medium with replenishment of 50% of the initial tamoxifen concentration as a standard procedure.

### Ammonium chloride treatment

Ammonium chloride was dissolved in EM and used at a final concentration of 10 mM. Ammonium chloride (NH_4_Cl) treatment is used to block lysosome acidification and therefore block autophagic flux. Treatment was for 4 h immediately prior to the collection of larvae for western blotting.

### Analysis of rod photoreceptor degeneration

Photoreceptor analysis was performed as described [19], with the following modifications. Photoreceptor counting was performed using the Fiji software (ImageJ). Briefly, the fluorescent photoreceptors were measured by selecting regions of interest after conversion to binary images. The total GFP-positive photoreceptor area measurement was used as a surrogate for total photoreceptor number.

### Statistical analysis

Data were analyzed using GraphPad 7.04 software. Multiple comparisons were calculated for statistical significance using one-way-ANOVA test followed by Tukey’s multiple comparisons test. Comparison of two conditions was performed using paired and unpaired t-tests, as appropriate. Data are presented as mean ± standard error the mean (SEM) or standard deviation (SD). P < 0.05 was considered significant.

## Supplementary Material

Supplemental Material

## References

[1] Rubinsztein DC, Bento CF, Deretic V. Therapeutic targeting of autophagy in neurodegenerative and infectious diseases. J Exp Med. 2015;212(7):979–990. 26101267 10.1084/jem.20150956 PMC4493419

[2] Dikic I, Elazar Z. Mechanism and medical implications of mammalian autophagy. Nat Rev Mol Cell Biol. 2018;19(6):349–364. 29618831 10.1038/s41580-018-0003-4

[3] Brand AH, Perrimon N. Targeted gene expression as a means of altering cell fates and generating dominant phenotypes. Development. 1993;118(2):401–415. 8223268 10.1242/dev.118.2.401

[4] Kawakami K, Asakawa K, Hibi M, et al. Gal4 driver transgenic zebrafish: powerful tools to study developmental biology, organogenesis, and neuroscience. Adv Genet. 2016;95:65–87. 27503354 10.1016/bs.adgen.2016.04.002

[5] Branda CS, Dymecki SM. Talking about a revolution: the impact of site-specific recombinases on genetic analyses in mice. Dev Cell. 2004;6(1):7–28. 14723844 10.1016/s1534-5807(03)00399-x

[6] Braun SM, Machado RA, Jessberger S. Temporal control of retroviral transgene expression in newborn cells in the adult brain. Stem Cell Reports. 2013;1(2):114–122. 24052947 10.1016/j.stemcr.2013.06.003 PMC3757750

[7] Gerety SS, Breau MA, Sasai N, et al. An inducible transgene expression system for zebrafish and chick. Development. 2013;140(10):2235–2243. 23633515 10.1242/dev.091520 PMC3640221

[8] Mizushima N. The ATG conjugation systems in autophagy. Curr Opin Cell Biol. 2020;63:1–10. 31901645 10.1016/j.ceb.2019.12.001

[9] Hu ZY, Chen B, Zhang JP, et al. Up-regulation of autophagy-related gene 5 (%B. J Biol Chem. 2017;292(44):18062–18074. 28928221 10.1074/jbc.M116.764795 PMC5672032

[10] Lopez A, Lee SE, Wojta K, et al. A152T tau allele causes neurodegeneration that can be ameliorated in a zebrafish model by autophagy induction. Brain. 2017;140(4):1128–1146. 28334843 10.1093/brain/awx005 PMC5382950

[11] Pyo JO, Yoo SM, Ahn HH, et al. Overexpression of Atg5 in mice activates autophagy and extends lifespan. Nat Commun. 2013;4(1):2300. 23939249 10.1038/ncomms3300 PMC3753544

[12] Nakatogawa H, Ishii J, Asai E, et al. Atg4 recycles inappropriately lipidated Atg8 to promote autophagosome biogenesis. Autophagy. 2012;8(2):177–186. 22240591 10.4161/auto.8.2.18373

[13] Fujita N, Hayashi-Nishino M, Fukumoto H, et al. An Atg4B mutant hampers the lipidation of LC3 paralogues and causes defects in autophagosome closure. Mol Biol Cell. 2008;19(11):4651–4659. 18768752 10.1091/mbc.E08-03-0312 PMC2575160

[14] Luo S, Rubinsztein DC. BCL2L11/BIM: a novel molecular link between autophagy and apoptosis. Autophagy. 2013;9(1):104–105. 23064249 10.4161/auto.22399 PMC3542209

[15] Luo S, Garcia-Arencibia M, Zhao R, et al. Bim inhibits autophagy by recruiting Beclin 1 to microtubules. Mol Cell. 2012;47(3):359–370. 22742832 10.1016/j.molcel.2012.05.040 PMC3419265

[16] Klionsky DJ, Abdel-Aziz AK, Abdelfatah S, et al. Guidelines for the use and interpretation of assays for monitoring autophagy (4th edition)(1). Autophagy. 2021;17:1–382. 33634751 10.1080/15548627.2020.1797280 PMC7996087

[17] Lopez A, Fleming A, Rubinsztein DC. Seeing is believing: methods to monitor vertebrate autophagy. Open Biol. 2018;8(10):180106. 30355753 10.1098/rsob.180106 PMC6223212

[18] Siddiqi FH, Menzies FM, Lopez A, et al. Felodipine induces autophagy in mouse brains with pharmacokinetics amenable to repurposing. Nat Commun. 2019;10(1):1817. 31000720 10.1038/s41467-019-09494-2 PMC6472390

[19] Moreau K, Fleming A, Imarisio S, et al. PICALM modulates autophagy activity and tau accumulation. Nat Commun. 2014;5(1):4998. 25241929 10.1038/ncomms5998 PMC4199285

[20] Bill BR, Petzold AM, Clark KJ, et al. A primer for morpholino use in zebrafish. Zebrafish. 2009;6(1):69–77. 19374550 10.1089/zeb.2008.0555 PMC2776066

[21] Sassen WAK R. A molecular toolbox for genetic manipulation of zebrafish. Adv Genom Genet. 2015;5:151–163.

[22] Akerberg AA, Stewart S, Stankunas K. Spatial and temporal control of transgene expression in zebrafish. PLoS One. 2014;9(3):e92217. 24643048 10.1371/journal.pone.0092217 PMC3958484

[23] Jacob J, Ribes V, Moore S, et al. Valproic acid silencing of ascl1b/Ascl1 results in the failure of serotonergic differentiation in a zebrafish model of fetal valproate syndrome. Dis Model Mech. 2014;7(1):107–117. 24135485 10.1242/dmm.013219 PMC3882053

[24] Gu Q, Yang X, Lv J, et al. AIBP-mediated cholesterol efflux instructs hematopoietic stem and progenitor cell fate. Science. 2019;363(6431):1085–1088. 30705153 10.1126/science.aav1749 PMC6469354

[25] Calzolari S, Terriente J, Pujades C. Cell segregation in the vertebrate hindbrain relies on actomyosin cables located at the interhombomeric boundaries. EMBO J. 2014;33(7):686–701. 24569501 10.1002/embj.201386003 PMC4000087

[26] Saitoh S, Maruyama T, Yako Y, et al. Rab5-regulated endocytosis plays a crucial role in apical extrusion of transformed cells. Proc Natl Acad Sci U S A. 2017;114(12):E2327–E36. 28270608 10.1073/pnas.1602349114 PMC5373379

[27] Sinha DK, Neveu P, Gagey N, et al. Photocontrol of protein activity in cultured cells and zebrafish with one- and two-photon illumination. Chembiochem. 2010;11(5):653–663. 20187057 10.1002/cbic.201000008

[28] Lu X, Agasti SS, Vinegoni C, et al. Optochemogenetics (OCG) allows more precise control of genetic engineering in mice with CreER regulators. Bioconjug Chem. 2012;23(9):1945–1951. 22917215 10.1021/bc300319c PMC3775343

[29] Feng Z, Nam S, Hamouri F, et al. Optical control of tumor induction in the Zebrafish. Sci Rep. 2017;7(1):9195. 28835665 10.1038/s41598-017-09697-x PMC5569104

[30] Goll MG, Anderson R, Stainier DY, et al. Transcriptional silencing and reactivation in transgenic zebrafish. Genetics. 2009;182(3):747–755. 19433629 10.1534/genetics.109.102079 PMC2710156

[31] Rafferty SA, Quinn TA. A beginner’s guide to understanding and implementing the genetic modification of zebrafish. Prog Biophys Mol Biol. 2018;138:3–19. 30032905 10.1016/j.pbiomolbio.2018.07.005

[32] De Paoli HC, Tuskan GA, Yang X. An innovative platform for quick and flexible joining of assorted DNA fragments. Sci Rep. 2016;6(1):19278. 26758940 10.1038/srep19278 PMC4725820

[33] Kawakami K, Abe G, Asada T, et al. zTrap: zebrafish gene trap and enhancer trap database. BMC Dev Biol. 2010;10(1):105. 20950494 10.1186/1471-213X-10-105 PMC2970601

[34] Scott EK, Baier H. The cellular architecture of the larval zebrafish tectum, as revealed by gal4 enhancer trap lines. Front Neural Circuits. 2009;3:13. 19862330 10.3389/neuro.04.013.2009 PMC2763897

[35] Tabor KM, Marquart GD, Hurt C, et al. Brain-wide cellular resolution imaging of Cre transgenic zebrafish lines for functional circuit-mapping. Elife. 2019;8(8):e42687. 30735129 10.7554/eLife.42687 PMC6392497

[36] Aizawa H, Goto M, Sato T, et al. Temporally regulated asymmetric neurogenesis causes left-right difference in the zebrafish habenular structures. Dev Cell. 2007;12(1):87–98. 17199043 10.1016/j.devcel.2006.10.004

[37] Venero Galanternik M, Nikaido M, Yu Z, et al. Localized Gene Induction by Infrared-Mediated Heat Shock. Zebrafish. 2016;13(6):537–540. 27057799 10.1089/zeb.2015.1161

[38] Shoji W, Sato-Maeda M. Application of heat shock promoter in transgenic zebrafish. Dev Growth Differ. 2008;50(6):401–406. 18430027 10.1111/j.1440-169X.2008.01038.x

[39] Auer TO, Duroure K, Concordet JP, et al. CRISPR/Cas9-mediated conversion of eGFP- into Gal4-transgenic lines in zebrafish. Nat Protoc. 2014;9(12):2823–2840. 25393779 10.1038/nprot.2014.187

[40] Kimura Y, Hisano Y, Kawahara A, et al. Efficient generation of knock-in transgenic zebrafish carrying reporter/driver genes by CRISPR/Cas9-mediated genome engineering. Sci Rep. 2014;4(1):6545. 25293390 10.1038/srep06545 PMC4189020

[41] Kimmel CB, Ballard WW, Kimmel SR, et al. Stages of embryonic development of the zebrafish. Dev Dyn. 1995;203(3):253–310. 8589427 10.1002/aja.1002030302

[42] Kwan KM, Fujimoto E, Grabher C, et al. The Tol2kit: a multisite gateway-based construction kit for Tol2 transposon transgenesis constructs. Dev Dyn. 2007;236(11):3088–3099. 17937395 10.1002/dvdy.21343

[43] Kimura S, Noda T, Yoshimori T. Dissection of the autophagosome maturation process by a novel reporter protein, tandem fluorescent-tagged LC3. Autophagy. 2007;3(9):452–460. 17534139 10.4161/auto.4451

