# Supplementary material for: An inducible expression system for the manipulation of autophagic flux in vivo
Source: Autophagy. 2022 Oct 30;19(5):1582–95. doi: 10.1080/15548627.2022.2135824 (PMC10240996; doi:10.1080/15548627.2022.2135824)
Supplement: Supplemental Material [file KAUP_A_2135824_SM4430.docx]

**Supplementary Information**

**Table S1.** Primer sequences.

| **attB1_atg5_ forward** | **GGGGACAAGTTTGTACAAAAAAGCAGGCTCAGCCACCATGATAATGGCAGATGACAAGG** |
| --- | --- |
| **attB2_atg5_reverse** | **GGGGACCACTTTGTACAAGAAAGCTGGGTAGAGAGCGGGAGGGTTAATGT** |
| **atg4b_forward** | **GGGATGGATGCAGCTACTCT** |
| **atg4b_reverse** | **CACAGTGGCCTTGAACGTC** |
| **bc12l11_forward** | **TTGGTTGTTGTCTTTATCGC** |
| **bcl2l11_reverse** | **CTGCTGGGTGTTCAATG** |
| **atg4b_C74A_forward** | **TGGGGCGCCATGCTTCGA** |
| **atg4b_C74A_reverse** | **CGCATCGAAGCATGGCGCC** |
| **bcl2l11_L128E_forward** | **TCGCTCGCGAAGAGCGAC** |
| **bcl2l11_L128E_reverse** | **GTCGCTCTTCGCGAGCGA** |
| **bcl2l11_F135E_forward** | **CATAGGCGATGAGGAGAATCGCCTC** |
| **bcl2l11_F135E_reverse** | **GAGGCGATTCTCCTCATCGCCTATG** |
| **attB1_FLAG_atg4b_forward** | **GGGGACAAGTTTGTACAAAAAAGCAGGCTCAATGGACTACAAAGACGATGACGACAAGGCCACCATGGATGCAGCTACTCT** |
| **attB1_His_bcl2l11_forward** | **GGGGACAAGTTTGTACAAAAAAGCAGGCTCAGCCACCATGCATCACCATCACCATCACTCCGGTCGATCTGACACGTCCAGAGAGCAA** |
| **attB2_M13F** | **GGGGACCACTTTGTACAAGAAAGCTGGGTAGTAAAACGACGGCCAGT** |
| **zflc3_forward** | **GTCCGGAATGCCTTCGGAAAAGAC** |
| **zflc3_reverse** | **GAGCTCCATGAGTGAAAGCCAATC** |


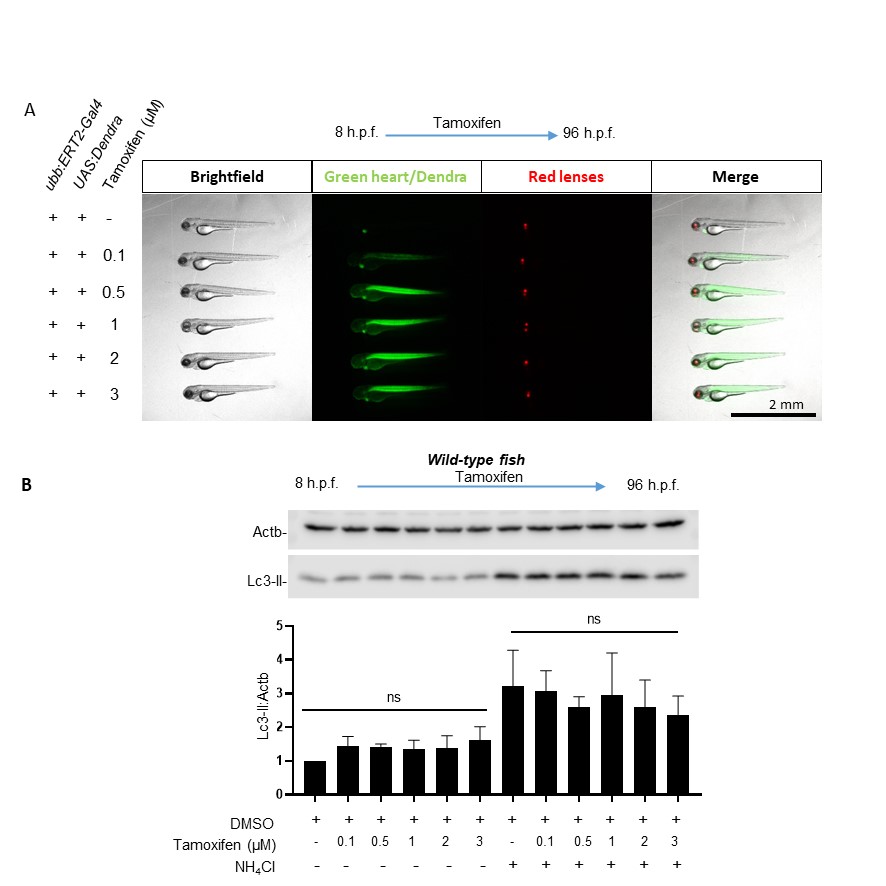


**Figure S1.** Tamoxifen concentration response. (**A**) Representative brightfield and fluorescence images of the offspring from crosses of *ubb:ERT2-Gal4* and *UAS:Dendra* showing the induction of transgene expression by tamoxifen at various concentrations (from 0.1 to 3 µM). Concentrations of tamoxifen equal or above 0.5 µM induce strong Dendra2 expression, resulting in green fluorescence across the whole fish compared to siblings treated with DMSO. Treatment with 0.1 µM tamoxifen resulted weak fluorescence and was not evident in all cells. No toxicity was observed at any concentration. Scale bar: 2 mm. (**B**) Wild-type larvae were treated with DMSO or 0.1 to 3 µM tamoxifen from 8 h.p.f. to 96 h.p.f. Four hours prior to tissue collection, each treatment group was divided in two and ammonium chloride added to one set. No significant differences in Lc3-II levels were observed between DMSO treated larvae and any concentration of tamoxifen either in basal or ammonium chloride treated groups demonstrating that tamoxifen treatment does not alter autophagic flux. N=5 independent experiments. Statistical analysis was performed using one-way-ANOVA test followed by Tukey’s multiple comparisons test; ns – not significant.

**
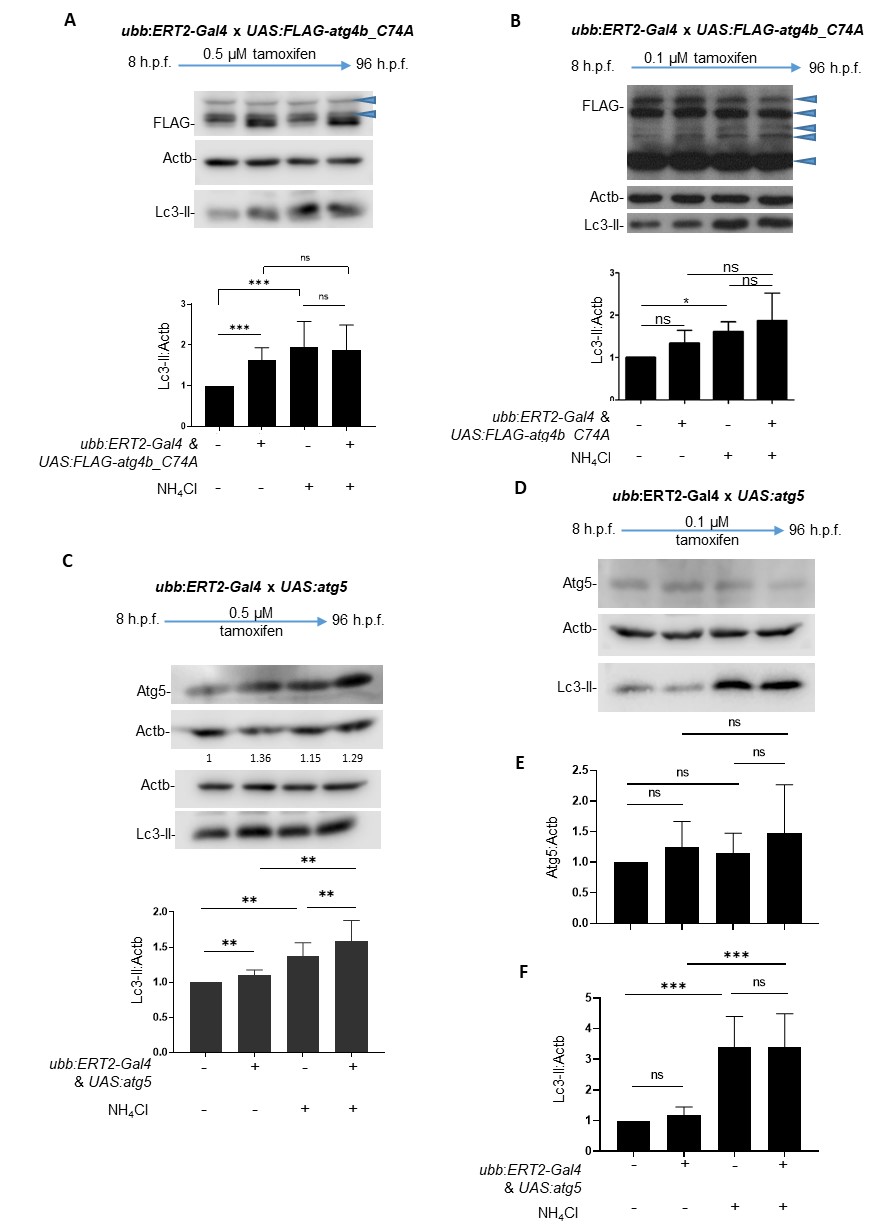
**

**Figure S2.** Determining the concentration of tamoxifen required to induce transgene expression and alter autophagic flux. (**A**) Induction of Atg4b^C74A^ expression with 0.5 µM tamoxifen results in a block in autophagic flux. All larvae from crosses of *ubb:ERT2-Gal4* with *UAS:atg4b_C74A* were treated with 0.5 µM tamoxifen from 8 h.p.f. to 96 h.p.f. Expression of FLAG-tagged Atg4b^C74A^ was observed in double transgenic larvae and correlated with an increase Lc3-II. Lc3-II levels do not increase further in NH_4_Cl treatment conditions indicating that Atg4b^C74A^ expression causes a block in autophagic flux. Non-specific bands (blue arrowheads) were observed in all treatment groups and genotypes in western blot for detection of the FLAG tag. (**B**) 0.1 µM tamoxifen treatment does not induce strong Atg4b^C74A^ expression and does not affect autophagic flux. All larvae from crosses of *ubb:ERT2-Gal4* with *UAS:atg4b_C74A* were treated with 0.1 µM tamoxifen from 8 h.p.f. to 96 h.p.f. Induction of the flag-tagged transgene could not be detected (high exposure shown; no bands are present at the size where the FLAG band would be detected). No significant differences in Lc3-II levels were observed in double transgenic larvae compared to non-transgenic siblings. As expected, ammonium chloride treatment resulted in a significant increase in Lc3-II in non-transgenic larvae. Non-specific bands (blue arrowheads) were observed in all treatment groups and genotypes in western blot for detection of the FLAG tag and partially overlap with the position that a positive FLAG signal would appear (e.g. as in Fig. S2A). The non-specific bands below the FLAG signal do not appear in all gels due to the position at which membranes were cut. (**C**) 0.5 µM tamoxifen treatment results in modest increases in autophagy induction in Atg5-expressing larvae. All larvae from crosses of *ubb*:*ERT2-Gal4* with *UAS:atg5* were treated with 0.5 µM tamoxifen from 8 h.p.f. to 96 h.p.f. Over-expression of Atg5 (relative to endogenous Atg5 levels) resulted in a significant increase in Lc3-II levels in basal conditions in double transgenic larvae compared to non-transgenic siblings. A further increase in Lc3-II levels was observed in ammonium chloride treatment conditions in double transgenic larvae compared to non-transgenic siblings, indicating that Atg5 overexpression resulted in an increase in autophagic flux. Due to differences in conditions needed for Atg5 immunoblotting, Atg5 and Lc3-II detection was performed on separate gels. The Actb loading control is presented for each gel and densitometry values are provided beneath the representative Atg5 blot. (**D-F**) 0.1 µM tamoxifen treatment does not induce strong Atg5 transgene expression and does not affect autophagic flux. All larvae from crosses of *ubb*:*ERT2-Gal4* with *UAS:atg5* were treated with 0.1 µM tamoxifen from 8 h.p.f. to 96 h.p.f. Atg5 remained at endogenous levels in all groups (quantified in **E**) and no significant differences in Lc3-II levels were observed in double transgenic larvae compared to non-transgenic siblings in either basal or ammonium chloride treatment conditions. In panels (**A**) to (**F**), graphs show mean values (± SEM) of densitometry of Lc3-II normalized to Actb (loading control) from 3 independent experiments. Statistical analysis was performed using paired t-test; ns – not significant; *p< 0.05; **p< 0.01; ***p<0.001.

**
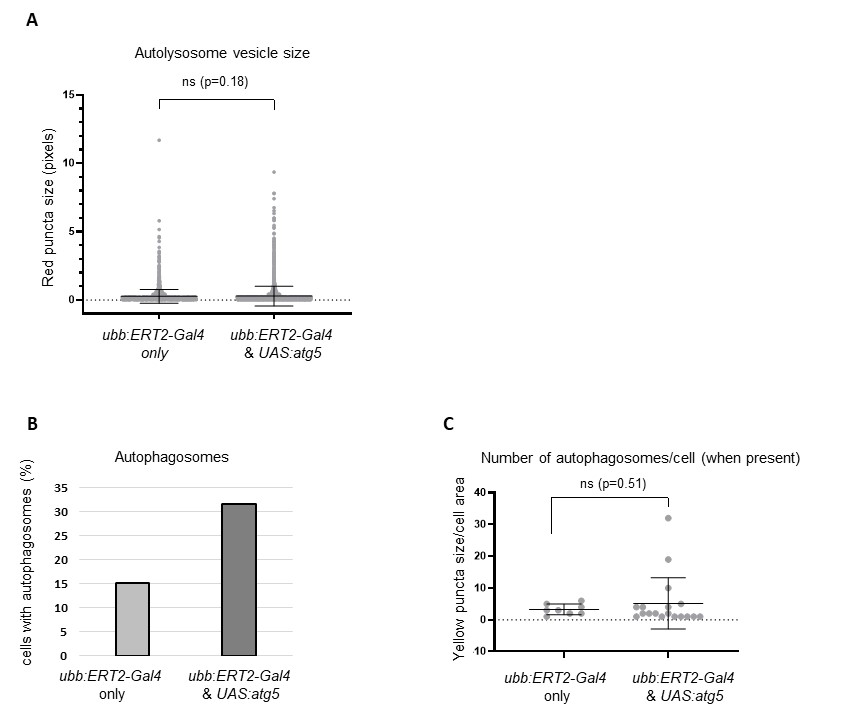
**

**Figure S3**. Assessing changes in autophagic flux *in vivo*. Eggs from a cross of *ubb*:*ERT2-Gal4* x *UAS:atg5* fish were injected with the *UAS:mRFP-GFP-lc3* reporter and the numbers of autophagosomes and autolysosomes were quantified in individual muscle cells of larvae at 96 h.p.f. (additional data to support Fig. 3). (**A**) Graph represents the size of individual red vesicles counted in muscle cells from control and Atg5-overexpressing larvae. Despite the increase in total number of red vesicles (see Fig.3), the overexpression of Atg5 did not induce any change in the size of red puncta compared to the control group. (**B**) Proportion of muscle cells with autophagosomes (yellow puncta). Despite the low numbers of autophagosomes visualized in both experimental groups, autophagosomes were more frequently seen in Atg5 overexpressing cells (31.6% of labelled muscle cells in atg5-expressing larvae vs. 15.1% of labelled muscle cells in control siblings). (**C**) Graph represents the number of autophagosomes per cell in the subpopulation of cells where autophagosomes where present (present in N= 8 non-transgenic and N= 18 Atg5 expressing cells). Atg5 overexpression did not increased the number of autophagosomes per cell (presented relative to the area of the cell in which puncta were observed).

**
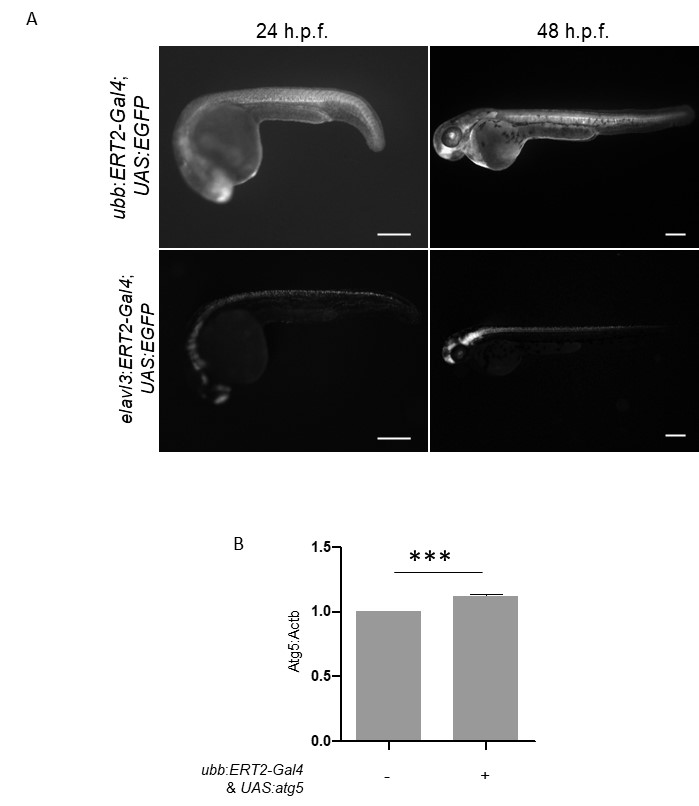
**

**Figure S4.** Spatial and temporal induction of transgene expression. (**A**) Larvae from crosses of *ubb*:*ERT2-Gal4* or *elavl3*:*ERT2-Gal4* and *UAS:EGFP* treated with 1 µM tamoxifen from 8 h.p.f. were imaged at 24 and 48 h.p.f. Larvae from crosses using the *ubb*:*ERT2-Gal4* driver showed robust, ubiquitous transgene expression at 24 h.p.f. and this remained at 48 h.p.f. Larvae from crosses using the *elavl3:ERT2-Gal4* driver showed expression of the transgene expression at 24 h.p.f. in the developing nervous system which became more evident and localised to the CNS 48 h.p.f. Representative images taken using GFP filter (excitation 395-455 nm; emission 480 nm). Scale bar represents 250 µm. (**B**) Quantification of induction of Atg5 expression upon late induction of expression (quantification of data presented in Fig. 4D).

**
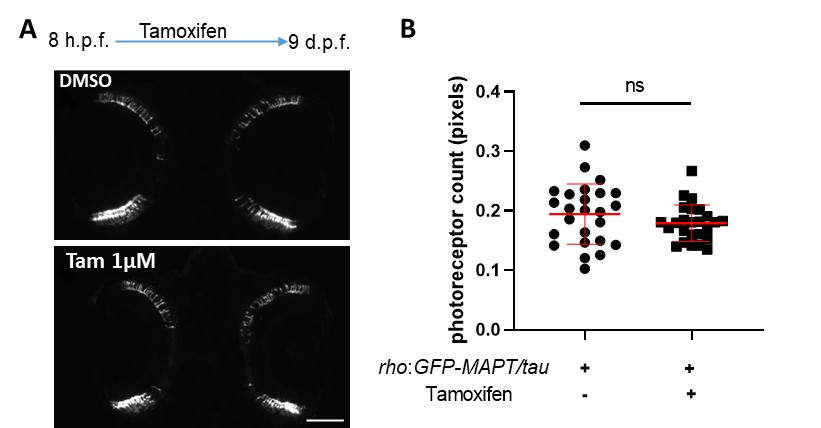
**

**Figure S5.** Tamoxifen treatment does not affect degeneration in a zebrafish model of tauopathy. (**A and B**) Representative images of cryosections across the central retina in *rho:GFP-MAPT/tau* fish treated with DMSO or 1 µM tamoxifen. Cryosections through the central retina (plane of section shown by blue line) were imaged (**A**) and quantified to determine the photoreceptor number and distribution (**B**). No differences were observed in the photoreceptor count indicating that tamoxifen treatment does not affect photoreceptor degeneration. Analysis performed on N=26 eyes. (**A**) Scale bar: 50 µm. (**B**) Statistical analysis was performed using unpaired t-test; ns – not significant.
